# Correlation Between Histopathology and Chemotherapy Response in Epithelial Ovarian Tumors

**DOI:** 10.7759/cureus.101088

**Published:** 2026-01-08

**Authors:** M Ramya, Surekha Talasila, T Sravanthi, M Srinivasulu

**Affiliations:** 1 Department of Surgical Oncology, Employee's State Insurance Corporation (ESIC) Medical College, Hyderabad, IND; 2 Department of Surgical Oncology, Gandhi Medical College, Secunderabad, IND; 3 Department of Surgical Oncology, MNJ Institute of Oncology and Regional Cancer Centre, Hyderabad, IND

**Keywords:** ca-125 kinetics, chemotherapy response score, epithelial ovarian carcinoma, histopathological subtype, interval debulking surgery, neoadjuvant carboplatin-paclitaxel, radiological response

## Abstract

Background: Epithelial ovarian carcinoma comprises biologically distinct histological subtypes with differing chemosensitivity; however, neoadjuvant carboplatin-paclitaxel is commonly administered using uniform treatment protocols.

Aim: This study aimed to determine whether histopathological subtype predicts multimodal response to neoadjuvant carboplatin-paclitaxel in advanced epithelial ovarian carcinoma.

Methods: In this prospective-retrospective observational study, 126 women with advanced epithelial ovarian carcinoma received platinum-taxane neoadjuvant chemotherapy followed by interval debulking surgery. Histological subtype, radiological response based on Response Evaluation Criteria in Solid Tumors (RECIST), serum cancer antigen 125 (CA-125) kinetics, omental and adnexal Chemotherapy Response Score (CRS 1-3), and extent of cytoreduction were recorded. Associations with histological subtype were analyzed using chi-square tests and analysis of variance (ANOVA).

Results: High-grade serous carcinoma (107/126, 84.9%) was the most common subtype, followed by low-grade serous carcinoma (12/126, 9.5%); mucinous, endometrioid, and clear cell tumors were infrequent. High-grade serous carcinomas demonstrated significant tumor shrinkage, marked decline in CA-125 levels, and predominantly CRS 2-3 responses. Low-grade serous and mucinous tumors showed minimal radiological regression, little change in CA-125, and uniformly CRS 1 responses. Endometrioid and clear cell tumors also showed CRS 1 responses but achieved favorable cytoreduction, although case numbers were very small. Optimal cytoreduction was achieved in 77/107 (72.0%) high-grade serous, 9/12 (75.0%) low-grade serous, 1/3 (33.3%) mucinous, and all endometrioid (3/3, 100.0%) and clear cell tumors (1/1, 100.0%). Neoadjuvant chemotherapy and surgery were well tolerated, with no treatment-related or perioperative mortality.

Conclusion: Histopathological subtype is a major determinant of response to neoadjuvant carboplatin-paclitaxel in epithelial ovarian carcinoma. Integrating RECIST response, CA-125 kinetics, CRS, and residual disease provides a pragmatic, histology-informed framework for treatment planning, particularly in settings where molecular profiling is limited.

## Introduction

Epithelial ovarian cancer remains the most lethal gynecologic malignancy, largely because most women present with advanced-stage disease and relapse despite initially high response rates to platinum-taxane chemotherapy [1]. Although conventional management often treats epithelial ovarian cancers as a uniform entity, extensive molecular and clinical evidence demonstrates that each histological subtype behaves as a distinct disease, with unique biological pathways and therapeutic vulnerabilities [2,3]. This heterogeneity is clinically relevant, as tumor subtype strongly influences chemosensitivity, patterns of spread, recurrence risk, and long-term survival.

Clear cell carcinoma exemplifies this biological divergence. Large multicenter retrospective cohorts demonstrate exceptionally poor sensitivity to platinum-based chemotherapy, with response rates usually low and markedly worse survival compared with serous carcinoma across all International Federation of Gynecology and Obstetrics (FIGO) stages [4,5]. Low-grade serous carcinoma displays similar resistance patterns due to mitogen-activated protein kinase (MAPK)-driven tumor biology, resulting in limited benefit from conventional chemotherapy and a shift toward endocrine and targeted therapies [6]. Conversely, high-grade serous carcinoma remains the most chemosensitive subtype, yet exhibits profound heterogeneity driven by DNA repair defects, BRCA1/BRCA2 (breast cancer susceptibility genes 1 and 2)-associated pathways, and adaptive resistance mechanisms [7,8]. Despite these well-characterized differences, frontline clinical decision-making often continues to rely on uniform chemotherapy protocols that do not explicitly incorporate histopathological subtype.

The development of the Chemotherapy Response Score (CRS) [9] represents a major advance in quantifying tumor regression following neoadjuvant chemotherapy. Studies have shown that a high CRS, indicating near-complete pathological response, is strongly associated with improved progression-free survival, reduced platinum resistance, and a higher likelihood of optimal cytoreduction [9-12]. However, these validations are overwhelmingly restricted to high-grade serous carcinoma, leaving substantial uncertainty regarding CRS performance across other histotypes. Furthermore, existing CRS studies rarely integrate radiological response patterns, cancer antigen 125 (CA-125) kinetics, or the influence of histological subtype on the achievability of interval cytoreduction, key determinants of survival outcomes. This lack of integrated, subtype-correlated response profiling represents a significant gap in real-world clinical evidence.

Contemporary reviews consistently emphasize the biological heterogeneity of epithelial ovarian cancers and strongly advocate for histology-informed therapeutic strategies [13-16]. However, no robust clinical study has systematically correlated histopathological subtype with multimodal response metrics, radiological tumor shrinkage, CA-125 kinetics, CRS across omental and adnexal specimens, and operability at interval surgery, in the setting of neoadjuvant carboplatin-paclitaxel chemotherapy. This evidence gap is particularly consequential in resource-constrained settings, where predictive molecular profiling is often limited and clinicians must rely on pragmatic, pathology-based biomarkers.

Against this background, the present study aims to determine whether histopathological subtype predicts response to neoadjuvant carboplatin-paclitaxel chemotherapy in epithelial ovarian carcinoma by correlating tumor subtype with radiological response, serum CA-125 kinetics, histopathological CRS in omental and adnexal specimens, and the likelihood of achieving optimal interval cytoreductive surgery. Generating such subtype-specific response data has the potential to refine prognostication, inform treatment planning, and support more personalized therapeutic strategies for women with advanced epithelial ovarian cancer.

## Materials and methods

Study design and setting

This was a prospective-retrospective, single-center observational study conducted in the Department of Surgical Oncology at MNJ Institute of Oncology and Regional Cancer Centre (MNJIO & RCC), Osmania Medical College, Hyderabad. The study protocol was approved by the Institutional Ethics Committee (IECC No: 20617001003D, dated 13/06/2021). The retrospective component included six patients treated immediately prior to ethics approval, whose data were retrieved from institutional records. The prospective component comprised 120 consecutive patients enrolled after ethics approval, all of whom provided written informed consent. Overall, consecutive patients with epithelial ovarian carcinoma planned for neoadjuvant chemotherapy (NACT) followed by interval debulking surgery (IDS) were included.

Patient selection

All women with proven ovarian malignancy who were planned for NACT followed by IDS at MNJIO & RCC were considered for recruitment. Inclusion criteria were (i) patients undergoing surgery for epithelial ovarian cancer, (ii) histologically confirmed epithelial ovarian malignancy, (iii) age >18 years, and (iv) willingness to participate and provide written informed consent. Patients were excluded if they received only non-surgical treatment (such as palliative chemotherapy), underwent primary cytoreductive surgery without prior NACT, or had non-epithelial ovarian tumors. Baseline evaluation included a detailed clinical history, physical examination, laboratory investigations, serum CA-125 measurement, and contrast-enhanced computed tomography (CECT) of the chest, abdomen, and pelvis.

Neoadjuvant chemotherapy protocol

All included patients received three to four cycles of platinum-taxane-based NACT. Chemotherapy consisted of intravenous carboplatin dosed at an area under the curve (AUC) of 5-6 on day 1, combined with paclitaxel 175 mg/m² administered intravenously on day 1. Cycles were repeated every three weeks, provided there was no unacceptable toxicity. Standard supportive care included dexamethasone premedication, antiemetics, and adequate hydration. Primary prophylaxis with recombinant granulocyte colony-stimulating factor was not routinely used. Serum CA-125 levels and CECT of the chest, abdomen, and pelvis were repeated after completion of the third cycle of NACT to assess treatment response and operability. A fourth cycle was administered in selected patients at the discretion of the treating multidisciplinary team when clinically indicated.

Interval debulking surgery

IDS was planned at least three weeks after the last cycle of NACT in patients deemed operable based on clinical and radiological assessment. Surgery was performed via midline laparotomy with maximal effort to achieve complete macroscopic cytoreduction (R0). Optimal cytoreduction was defined as residual disease <1 cm in maximum diameter, although every attempt was made to remove all visible tumor deposits. The standard surgical procedure included total abdominal hysterectomy, bilateral salpingo-oophorectomy, and omentectomy in all cases. Additional procedures, such as anterior resection, diaphragmatic or peritoneal stripping, and appendicectomy, were performed when required to achieve optimal or complete cytoreduction. Final residual disease status (R0, optimal <1 cm, or suboptimal ≥1 cm) was recorded for correlation with histological subtype and other response parameters. Two weeks after surgery, patients were planned for three additional cycles of adjuvant carboplatin-paclitaxel chemotherapy using the same dosing schedule as the neoadjuvant phase, subject to postoperative recovery and fitness for systemic therapy.

Response assessment

Radiological Response

Baseline tumor burden was documented on pre-NACT CECT, and radiological response was reassessed after three cycles of chemotherapy using the same imaging modality. Response was categorized according to Response Evaluation Criteria in Solid Tumors (RECIST) [17] as follows: complete response (CR), disappearance of all target lesions with pathological lymph nodes measuring <10 mm in short axis; partial response (PR), at least a 30% decrease in the sum of diameters of target lesions compared with baseline; progressive disease (PD), at least a 20% increase in the sum of diameters of target lesions compared with the smallest recorded sum (including baseline if smallest), with an absolute increase of ≥5 mm or the appearance of new lesions; and stable disease (SD), insufficient change to qualify as PR or PD. For analysis, patients with CR or PR were classified as radiological responders, while those with SD or PD were considered non-responders. These categories were used to examine associations with histopathological subtype, CA-125 kinetics, CRS, and surgical cytoreduction status.

Serum CA-125 Kinetics

Serum CA-125 was measured at baseline (pre-NACT) and again after three cycles of NACT, concurrent with radiological reassessment. CA-125 kinetics were expressed as absolute values at each time point and as a change from baseline to post-NACT. These parameters were correlated with histopathological subtype, radiological response (responder vs non-responder), CRS in omental and adnexal specimens, and the ability to achieve optimal or complete cytoreduction at IDS.

Histopathology and Chemotherapy Response Score

All patients had histological confirmation of epithelial ovarian carcinoma prior to or at the time of IDS. Histopathological subtype (e.g., high-grade serous, low-grade serous, clear cell, and other epithelial subtypes, as applicable) was assigned according to standard diagnostic criteria and served as the primary stratification variable for analysis.

At IDS, representative tissue samples from omental and adnexal sites were submitted for detailed histopathological evaluation. Omental and adnexal slides were jointly reviewed by pathologists, and each site was assigned a Chemotherapy Response Score (CRS), adapted from Böhm et al. [9], using a three-tier system (CRS 1-3) reflecting the degree of tumor regression following NACT. CRS 1 indicated minimal or no response, CRS 2 indicated appreciable but incomplete response, and CRS 3 indicated near-complete or complete pathological response. The number of slides examined for each site was recorded. CRS at each site was correlated with histopathological subtype, radiological response, CA-125 kinetics, and surgical cytoreduction outcomes.

Statistical analysis

Data were summarized as mean ± standard deviation for continuous variables and as frequencies and percentages for categorical variables. Continuous variables (including age, CA-125 levels, and changes in CA-125) were compared across histopathological subtypes or CRS categories using one-way analysis of variance (ANOVA), as appropriate. Categorical variables, including histological subtype, radiological response status, CRS category (1-3), and cytoreduction status (R0/optimal/suboptimal), were compared using the Pearson chi-square test. Histopathological subtype was treated as the principal independent variable, and its association with radiological response, CA-125 kinetics, CRS (omentum and adnexa), and residual disease status was examined. Statistical significance was defined as a two-sided p-value < 0.05. All analyses were performed using IBM SPSS Statistics for Windows, Version 21.0 (Released 2012; IBM Corp., Armonk, NY, USA).

## Results

A total of 126 women with advanced epithelial ovarian carcinoma who received neoadjuvant carboplatin-paclitaxel chemotherapy followed by interval debulking surgery were included in the analysis.

Baseline clinicopathologic profile

High-grade serous carcinoma was the predominant histological subtype (107/126, 84.9%), followed by low-grade serous carcinoma (12/126, 9.5%), while mucinous, endometrioid, and clear cell tumors comprised small subgroups. High-grade serous carcinomas occurred at an older mean age (56.1 years), whereas low-grade serous and mucinous tumors were observed in younger women (mean age ~40 years). Most patients with high-grade serous carcinoma were postmenopausal (88/107, 82.2%), while low-grade serous, mucinous, and clear cell tumors were restricted to premenopausal women; endometrioid carcinomas were seen in both pre- and postmenopausal groups (Table 1).

**Table 1:** Baseline clinicopathologic characteristics by histological subtype (n = 126)

Histological subtype	n	%	Mean age (years)	Premenopausal, n	Postmenopausal, n
High-grade serous	107	84.9	56.1	19	88
Low-grade serous	12	9.5	40.6	12	0
Mucinous	3	2.4	39.0	3	0
Endometrioid	3	2.4	50.0	1	2
Clear cell	1	0.8	52.0	1	0
Total	126	100.0	-	36	90

Neoadjuvant chemotherapy delivery

All patients completed the planned neoadjuvant chemotherapy before interval debulking surgery. The majority received six cycles of platinum-taxane NACT (112/126, 88.9%), while 9/126 (7.1%) and 5/126 (4.0%) patients received three and four cycles, respectively, according to performance status and multidisciplinary team decisions.

Radiological response

Baseline primary tumor size was the largest in high-grade serous and mucinous carcinomas. After NACT, high-grade serous, clear cell, and endometrioid tumors showed clear radiological shrinkage, whereas low-grade serous and mucinous carcinomas exhibited minimal or no reduction in size. The reduction in tumor size was statistically significant for high-grade serous carcinoma compared with the other histological subtypes (p = 0.0001) (Table 2).

**Table 2:** Mean primary tumor size before and after neoadjuvant chemotherapy

Histological subtype	Pre-NACT size (cm)	Post-NACT size (cm)
High-grade serous	13.0	6.9
Low-grade serous	8.2	8.0
Mucinous	11.3	11.5
Clear cell	10.0	7.0
Endometrioid	10.0	7.3

Biochemical response: CA-125 kinetics

High-grade serous carcinoma had the highest baseline CA-125 levels (mean 1677 U/mL) and exhibited the most pronounced biochemical response, with a marked post-NACT decline to 148 U/mL. Low-grade serous, mucinous, and clear cell tumors showed only modest decreases in CA-125, reflecting their relative chemoresistance, whereas endometrioid carcinomas demonstrated a substantial reduction despite small numbers (296 to 38.7 U/mL). Overall, the pattern of CA-125 kinetics closely paralleled radiological response across histological subtypes (Table 3).

**Table 3:** Mean CA-125 levels before and after neoadjuvant chemotherapy

Histological subtype	Pre-chemotherapy CA-125 (U/mL)	Post-chemotherapy CA-125 (U/mL)
High-grade serous	1677	148
Low-grade serous	415	403
Mucinous	147	125
Clear cell	349	315
Endometrioid	296	38.7

Pathological response (Chemotherapy Response Score)

Among high-grade serous carcinomas (n = 107), CRS 1, CRS 2, and CRS 3 were observed in 11, 61, and 35 cases, respectively, indicating appreciable or near-complete pathological regression in the majority. In contrast, all low-grade serous, mucinous, clear cell, and endometrioid tumors exhibited only CRS 1 changes in both omentum and adnexa, reflecting minimal histological response. CRS was significantly higher in high-grade serous compared with low-grade serous carcinoma (p < 0.0001), and higher CRS categories qualitatively corresponded with greater CA-125 decline and radiological tumor shrinkage (Table 4).

**Table 4:** Chemotherapy Response Score (CRS) distribution by histological subtype The p-value denotes the overall association between histological subtype and CRS category (CRS1 3), assessed using the Pearson chi-square test; p < 0.0001 (two-sided).

Histological subtype	n	CRS1, n (%)	CRS2, n (%)	CRS3, n (%)
High-grade serous	107	11 (10.3%)	61 (57.0%)	35 (32.7%)
Low-grade serous	12	12 (100.0%)	0 (0.0%)	0 (0.0%)
Mucinous	3	3 (100.0%)	0 (0.0%)	0 (0.0%)
Clear cell	1	1 (100.0%)	0 (0.0%)	0 (0.0%)
Endometrioid	3	3 (100.0%)	0 (0.0%)	0 (0.0%)

Cytoreductive surgery outcomes

All 126 women underwent interval debulking surgery. Optimal cytoreduction (<1 cm residual disease) was achieved in 77/107 (72.0%) high-grade serous and 9/12 (75.0%) low-grade serous tumors, but in only one-third of mucinous carcinomas (1/3), whereas all endometrioid (3/3) and clear cell (1/1) tumors achieved optimal debulking. The difference in optimal cytoreduction rates between high- and low-grade serous carcinoma was statistically significant (p = 0.013). Across the cohort, patients with higher CRS (particularly CRS 3) were more likely to achieve optimal or complete cytoreduction, consistent with superior radiological and biochemical responses (Table 5).

**Table 5:** Extent of cytoreduction by histological subtype

Histological subtype	Optimal cytoreduction, n (%)	Suboptimal cytoreduction, n (%)
High-grade serous	77 (72.0%)	30 (28.0%)
Low-grade serous	9 (75.0%)	3 (25.0%)
Mucinous	1 (33.3%)	2 (66.7%)
Endometrioid	3 (100.0%)	0 (0.0%)
Clear cell	1 (100.0%)	0 (0.0%)

Treatment-related toxicity and postoperative complications

Neoadjuvant chemotherapy was generally well tolerated, with no grade 4 toxicities or chemotherapy-related deaths. Fatigue and alopecia occurred in all patients (126/126, 100.0%), while anemia, nausea/vomiting, taste changes, and other hematological toxicities were common but manageable. Postoperative complications were infrequent and predominantly minor, including ileus, atelectasis, and wound infection; no perioperative deaths were reported (Table 6, Table 7).

**Table 6:** Complications of neoadjuvant chemotherapy (n = 126)

Complication	n (%)
Anemia	88 (69.8%)
Thrombocytopenia	32 (25.4%)
Neutropenia	30 (23.8%)
Nausea and vomiting	82 (65.1%)
Diarrhea	8 (6.3%)
Peripheral neuropathy	13 (10.3%)
Alopecia	126 (100.0%)
Allergic reaction	3 (2.4%)
Taste change	110 (87.3%)
Fatigue	126 (100.0%)
Mortality	0 (0.0%)

**Table 7:** Postoperative complications (n = 126)

Complication	n (%)
Wound infection	4 (3.2%)
Ileus	10 (7.9%)
Atelectasis	8 (6.3%)
Urinary tract infection	1 (0.8%)
Mortality	0 (0.0%)

Taken together, high-grade serous carcinoma exhibited the most consistent multimodal response (radiological shrinkage, CA-125 decline, higher CRS, and favorable cytoreduction rates), whereas low-grade serous and mucinous tumors remained largely chemoresistant across all metrics, and endometrioid and clear cell tumors demonstrated favorable but numerically limited response patterns.

## Discussion

In this single-center cohort, histopathological subtype clearly emerged as a major determinant of response to neoadjuvant carboplatin-paclitaxel in advanced epithelial ovarian carcinoma. High-grade serous carcinoma exhibited the most favorable multimodal profile, characterized by marked radiological shrinkage, profound declines in CA-125, and a predominance of CRS 2-3, resulting in high rates of optimal cytoreduction. These findings align with the recognized chemosensitivity of high-grade serous carcinoma, driven by homologous recombination defects and TP53-mutant biology [2,3,7], and mirror prior CRS validation studies showing that CRS 3 identifies a subgroup with superior progression-free outcomes and lower risk of primary platinum resistance [9-12]. In the present study, progression-free survival and overall survival were not evaluated; therefore, CRS, radiological response, CA-125 kinetics, and cytoreduction status are interpreted as surrogate measures of treatment sensitivity and operability rather than direct survival endpoints.

By contrast, low-grade serous and mucinous tumors in our cohort exhibited minimal radiological shrinkage, flat CA-125 kinetics, and uniformly low CRS, despite technically successful surgery. This pattern is consistent with the literature describing low-grade serous carcinoma as intrinsically chemoresistant, MAPK-driven disease, where endocrine therapy and mitogen-activated protein kinase kinase (MEK) inhibition are more effective than platinum-taxane regimens, and primary mucinous carcinoma as similarly poorly responsive to standard ovarian protocols [2,4-6,14]. The observed CA-125 decline, CRS 1 pattern, and universal optimal cytoreduction in the small endometrioid and clear cell subgroups should therefore be interpreted cautiously, given the very limited numbers and the known long-term risk of relapse in clear cell carcinoma despite aggressive surgery [3-5].

Our results support current evidence that appropriately selected patients benefit from neoadjuvant carboplatin-paclitaxel followed by interval debulking, which increases the likelihood of optimal cytoreduction without compromising survival and with acceptable perioperative morbidity [1,18]. Importantly, they also show that CRS and CA-125 kinetics are not “one-size-fits-all” biomarkers but are strongly influenced by histological subtype, reinforcing calls for histology-informed algorithms when deciding on maintenance strategies such as poly(ADP-ribose) polymerase (PARP) inhibition or bevacizumab, and when considering early integration of endocrine or targeted therapy in chemoresistant subtypes [13,14,19,20].

The strengths of this study include uniform NACT and surgical protocols, systematic CRS scoring of both omental and adnexal specimens, and integrated analysis of radiological, biochemical, and pathological response by subtype. Key limitations are its single-center design, predominance of high-grade serous carcinoma, very small numbers of non-serous tumors, lack of BRCA1/2, homologous recombination deficiency (HRD), and other molecular profiling, and absence of analyzed survival endpoints, including progression-free survival and overall survival, as well as limited mature follow-up data [15,16].

## Conclusions

These findings reinforce that epithelial ovarian carcinoma comprises biologically distinct diseases with differing chemosensitivity profiles. High-grade serous carcinoma derives substantial benefit from neoadjuvant carboplatin-paclitaxel, with high CRS rates and favorable cytoreduction, whereas low-grade serous and mucinous tumors remain largely chemoresistant. Incorporating histological subtype into response assessment using a simple, multimodal framework (RECIST response, CA-125 kinetics, CRS, and residual disease) may provide a pragmatic pathway toward more personalized, histology-driven treatment decisions, particularly in settings where comprehensive molecular testing is not routinely available.
